# Determination of Volatile Terpenes in Coriander Cold Pressed Oil by Vacuum Assisted Sorbent Extraction (VASE)

**DOI:** 10.3390/molecules26040884

**Published:** 2021-02-08

**Authors:** Henryk H. Jeleń, Monika A. Marcinkowska, Maria Marek

**Affiliations:** 1 Faculty of Food Science and Nutrition, Poznań University of Life Sciences, Wojska Polskiego 31, 60-624 Poznań, Poland; monika.marcinkowska@up.poznan.pl (M.A.M.); maria.marek@gal.com.pl (M.M.); 2 GAL, Krótka 4, 61-012 Poznań, Poland

**Keywords:** cold pressed oils, coriander oil, VASE, terpenes, volatile compounds

## Abstract

Cold-pressed plant oils are of high interest to consumers due to their unique and interesting flavors. As they are usually only pressed at low temperatures and filtered, without further processing stages (as refining), they preserve their character that originates from the plant the oil was extracted from. Coriander cold pressed oil is gaining popularity as a novel product, obtained from its fruits/seeds; due to the high amount of terpenes, it has very characteristic flavor. A novel, vacuum-assisted sorbent extraction (VASE) method was used to extract terpenes from coriander cold pressed oil. Optimal parameters were determined. The profile of compounds extracted using VASE was compared with that of classic hydrodistillation method. Moreover, 17 monoterpene hydrocarbons and alcohols were identified with β-linalool as the main compound, followed by α-pinene, γ-terpinene, camphor, sylvestrene, β-pinene, and o-cymene. Differences were noted between profiles of terpenes after hydrodistillation and VASE extraction. For 8 out of 17 terpenes, VASE was used for their quantitative analysis. Regarding simplicity of the method, small sample requirement (200 mg) and short extraction time (5 min), VASE combined with GC/MS is well suited for characterization of terpenes in such matrix as plant oils.

## 1. Introduction

Coriander (*Coriandrum sativum* L.) is an annual herb of the Apiaceae family that is used in many industries. It is a culinary and medicinal plant, native to the Near East and Mediterranean area, but it has spread to India and China. Nowadays, coriander is a common herb around the world [1]. Coriander has a number of therapeutic properties (e.g., antibacterial, anticancer, antidiabetic, antimutagenic, antioxidant, anti-inflammatory, analgesic, anticonvulsant, blood pressure-lowering, cholesterol-lowering, and sedative activities [2,3]) desired in the medical and pharmaceutical industries, due to the presence of specific metabolites. Coriander has been used in traditional medicine for ages to counteract several disorders, such as dysentery, dyspepsia, giddiness spasm, neuralgia, and gastric complaints [4]. Moreover, coriander, overall, is used as a spice. Due to its spicy citrus flavor [5], it is also used as a flavoring agent in food products and in the perfume and cosmetic industries. All of the above applications make coriander an economically important plant [6].

Essential oils are aromatic and volatile oily liquids extracted from different plant tissues, namely flowers, seeds, leaves, twigs, root, fruit, etc. The compounds forming the essential oils are accumulated in diverse secretory glands and trichomes in plants; however, most common essential oils are obtained from seeds and flowers. Essential oils can be gained from many materials of plant origin; therefore, their mixture consists mainly of specific metabolites, mainly terpenoids. The chemical nature of these compounds makes them less soluble in polar solvents, while they are soluble in non-polar solvents, such as methylene chloride or hexane [7].

The extraction step is crucial in isolating the desired components from a complex biological matrix. There are conventional methods, including hydrodistillation, cold-pressed extraction, and, sometimes, solvent-based extraction; however, their greatest disadvantage is the time-consuming and low efficiency extraction, which results in high production costs. On the other hand, it is possible to use modern extraction methods (e.g., enzyme-assisted extraction, enzyme digestion, microwave-assisted extraction, pressurized liquid extraction, pulsed electric field extraction, supercritical fluid extraction, ohmic heating, and ultrasound-assisted extraction) that reduce the limitations of conventional methods. Nevertheless, supplying energy to the extraction system reduces target thermolabile biocomponents. The use of cold press is therefore not only an eco-friendly method, but also has an advantage over unconventional extraction methods in terms of product quality [8].

Coriander seeds contain more than 1% of essential oil, with the major components being linalool (more than 50%), geranyl acetate, borneol, p-cymol, α-pinene, bornyl acetate, desilaldehyde, citronellol, and thymol [9,10]. The coriander seed contains plant oil, which is a rich source of the rare monounsaturated isomer of oleic acid, namely petroselinic acid (C18:1n12) [11]. Coriander oil has a pleasant odor with odor descriptors, such as floral, turpentine-like, pleasant, green, herbal, cooling, earthy, spicy, sweet, and rose-like [12]. Since 2013, coriander seed oil has been a novel food ingredient, according to the European Commission, and can be used as a food supplement [11].

Headspace analysis is a routine extracting method of food product samples with a number of advantages. The most popular method of extracting volatile compounds from food is solid-phase microextraction (SPME); however, the most important limitation of this method is the difficult extraction of compounds with low Henry’s law volatility constants. Vacuum assisted sorbent extraction (VASE) is a novel approach for headspace sorbent extraction and only a few papers have been published demonstrating the potential of the technique. Application of vacuum increases the yield of the extraction process of compounds that are difficult to extract, with the use of commercialized sorbent traps [13]. The sorbent traps are filled with a significant amount of Tenax (equivalent to approximately 500 times the volume typically used for SPME), which additionally supports exhaustive extraction [14]. Moreover, as the traps used in VASE are closed and vacuum is the driving force for extraction, there are no issues related to breakthrough volumes often present for purge and trap methods and other forms of dynamic headspace.

The main goal of the present investigation was to study the profile of volatile terpenes in coriander cold-pressed seed oil by VASE and explore its possible potential to extract the flavor compounds from plant oils.

## 2. Results

### 2.1. Chemical Characteristics of Coriander Oil

Oil was obtained from coriander fruit and was pressed using a screw press at temperatures not exceeding 40 °C, under nitrogen atmosphere. Oil used in the experiments was characterized by a peroxide value of 2.86 ± 0.3 meq O2/kg, free fatty acids (FFA) = 2.0 mg KOH/g, and the following fatty acids methyl esters (FAME) profile: C16:0–3.89% (*w*/*w*), C16:1–0.13%, C18:0–1.65%, C18:1–73.79%, C18:2–15.91%, C18:3–0.24%, C20:0–0.25%. As the column used for FAME analysis was not able to resolve oleic acid methyl ester (C18:1 n-9) from petroselinic acid (C18:1 n-12), both isomers were listed under C18:1. The characteristics of cold pressed coriander oil fulfilled the regulations on novel foods, Commission Implementing Regulation (EU) 2019/2165, except for the exceeded stearic acid contents (1.65% instead of <1.5%).

### 2.2. Identification of Terpenes Using VASE

**Figure 1:**
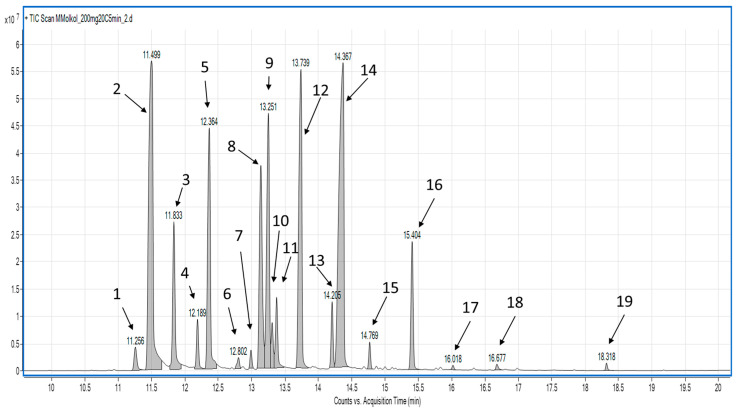
TIC chromatogram of main volatile terpenes obtained using vacuum-assisted sorbent extraction (VASE) at 20 °C. (1) α-thujene; (2) α-pinene; (3) camphene; (4) sabinene; (5) β-pinene; (6) α-phellandrene; (7) α-terpinene; (8) o-cymene; (9) sylvestrene; (10) unknown; (11) cis-ocimene; (12) γ-terpinene; (13) terpinolene; (14) β-linalool; (15) allo-ocimene; (16) camphor; (17) unknown; (18) geraniol; (19) geranyl acetate.

The characteristic aromatic feature of coriander cold pressed oil is its unique aroma. The aroma of coriander oil is associated with a high amount of terpenes present in it, which are transferred to the oil during pressing. VASE allowed for extraction of terpenes present in coriander oil. Due to the nature of the matrix (oil composed mainly of triglycerides), headspace analysis, such as VASE, is the most convenient (and fastest) type of analysis to identify (and quantify) volatiles in it. Typical total ion chromatogram (TIC) obtained using VASE is presented on Figure 1. A total of 17 main terpenes were identified using VASE extraction at 20 °C. The most abundant compound extracted was β-linalool, which was 23.05% of the total peak area (Table 1), followed by α-pinene (17.94%), γ-terpinene (13.96%), sylvestrene (10.20%), β-pinene (9.70%), and o-cymene (8.93%)—regarding compounds present in >5%. Eight compounds were identified based on their retention indices and mass spectra compared to NIST 02 library, and for nine terpenes, authentic standards were used for their identity confirmation (Table 1).

### 2.3. Determination of Optimal Parameters for VASE Extraction

VASE is a sorbent-based technique and requires experimental elaboration of optimal extraction parameters, which would allow for sufficient sensitivity and separation of extracted compounds. For the selection of best extraction parameters, various sample sizes, extraction temperatures, as well as extraction times were tested. For the analysis of terpenes in coriander oil, the sensitivity was not an issue, as the amount of terpenes is very high in this oil. The majority of the problems were related to column overloading and insufficient peak separation, as the VASE desorption unit used a constant split frit.

**Figure 2:**
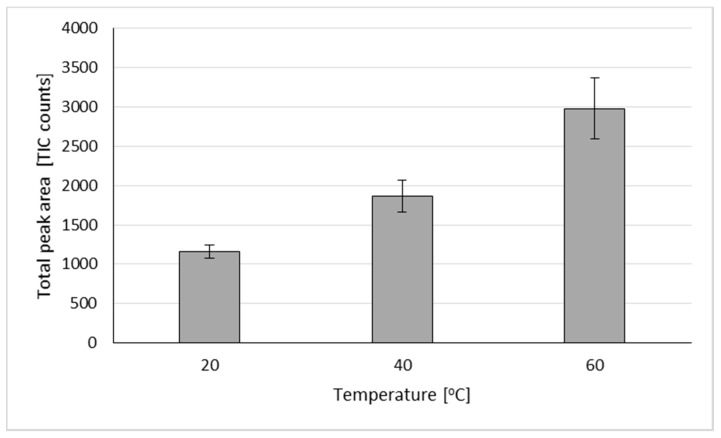
Total peak areas of terpenes extracted from cold pressed coriander oil using VASE at different extraction temperatures. All extractions were performed for 5 min; total peak areas expressed in million counts.

In the first step, an optimal sample size was chosen after analysis of 2000 mg, 1000 mg, and 200 mg samples. Analyses were initially run for 20 min at the temperature of 60 °C for various sample sizes. Chromatograms illustrating extraction parameters are shown in the supplementary files (Figures S1–S3). From the initial conditions, a sample size of 200 mg was chosen for further experiments (Figure S1). Decreasing further sample sizes may result in worse repeatability and reproducibility. In the next step, the temperature of extraction was checked, and from the initial ones tested (60 °C, 40 °C, and 20 °C), the last one was chosen, based on the quality of chromatograms (Figure S2). The choice of temperature is highly dependent on the compounds (which are subjects of extraction). The coriander terpenes consisted mainly of monoterpene hydrocarbons, as well as monoterpene alcohols, among which, linalool dominated. As can be seen in Figure 2, temperature had a significant effect on the extraction of analyzed terpenes from coriander oil. The total peak area was almost three times higher for 60 °C compared to 20 °C. The most significant difference, in terms of compound identification, was noted for two compounds with very low volatility—geraniol and geranyl acetate. Their peak areas were approximately four times bigger for 60 °C extraction compared to 20 °C extraction (Table 1).

In the final step, different extraction times were tested (3 min, 5 min, 10 min, and 20 min) to select the one providing the best peak separation with sufficient sensitivity. For the tested extraction times, 5 min was selected as the one providing good separation and high peak areas (Figure S3).

### 2.4. Comparison of Volatile Terpene Profiles Obtained by VASE and Hydrodistillation

**Figure 3:**
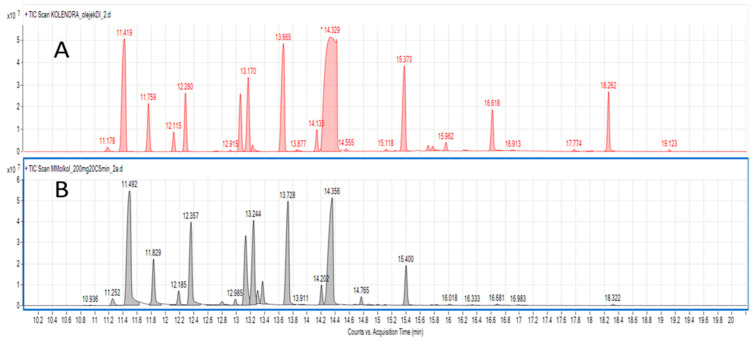
TIC chromatograms of coriander terpenes obtained after hydrodistillation of coriander cold pressed oil (**A**) and after VASE extraction (**B**).

Hydrodistillation is a standard extraction method for isolation of essential oils from plants and was used in almost all references on coriander essential oils research. For our analytical purposes, VASE was performed from cold pressed oil obtained from coriander fruit. To relate the results obtained using VASE with hydrodistillation results, the latter process was performed for cold pressed oil. Oil and water mixture (1:4) was subjected to hydrodistillation in a Deryng apparatus for 2 h, which allowed collection of essential oil for comparison. Figure 3 and Table 1 show terpene profile comparison of hydrodistilled compounds vs. those obtained by VASE extraction. The most striking difference is the high percentage (%) amount of β-linalool in hydrodistilled oil compared to VASE (almost 45% compared to 23–27%). Moreover, other monoterpene alcohol—geraniol and its acetate—was in higher concentration in hydrodistilled oil. Camphor, with the vapor pressure of 0.22 mmHg and logP value of 2.38, was also extracted with more efficiency using hydrodistillation. On the other hand, for almost all monoterpene hydrocarbons (with the exception of α-pinene) their percentage (%) amount in VASE extracts was higher than for hydrodistillation. For some compounds, the differences were 2-fold (sylvestrene) up to 10-fold (allo-ocimene). Interestingly, structurally related isomers of α-pinene and β-pinene behaved differently in both types of extraction. Data on comparison of terpene profiles in Table 1 indicate the profound differences related to the nature of extraction.

### 2.5. Terpenes Quantitative Analysis Using VASE

VASE can be used for profiling volatile compounds; however, it can also be used for quantitative analyses, bearing in mind the partition coefficient nature of extraction. With direct analysis (injection) of essential oils, an approximation can be assumed regarding response factors of particular compounds, especially when quantitation is made using an flame ionization (FID) detector. In case of headspace analysis, calibration should be performed for every analyte using separate standards. For quantitation of constituents of coriander oil, the following eight standards were used: α-pinene, camphene, β-pinene, α-terpinene, γ-terpinene, terpinolene, β-linalool, and camphor. Standard curves were prepared in refined rapeseed oil in a concentration range of 0.32–10.4 g/L. Calibration curves were linear in this range (R^2^ > 0.994) for all compounds but α-terpinene, for which the logarithmic curve was created (Figure S4). For compounds quantified using standard curves, the following concentrations were obtained (g/L of coriander cold pressed oil): α-pinene—6.50 ± 0.54; camphene—1.91 ± 0.31; β-pinene—4.89 ± 0.65; α-terpinene—3.17 ± 0.26; γ-terpinene—4.61 ± 0.43; terpinolene—0.92 ± 0.16; β-linalool—43.55 ± 4.28; and camphor—1.70 ± 0.26. For quantified compounds, no LODs were estimated, as the sensitivity of the method is not an issue when analyzing, at least, the main terpenes in coriander oil. However, for samples representing 0.020 g/L of each analyte, the s/n ratios for TIC ranged between 14 (camphene and γ-terpinene) and 36 (α-terpinene). Moreover, repeatability for all compounds that were measured as TIC peak areas of particular identified compounds was generally < 10% relative standard deviation (RSD) for all compounds. These results show the potential of the method; however, analysis of all detected compounds using their standards would provide a complete picture of the method’s quantitative performance.

## 3. Discussion

**Figure 4:**
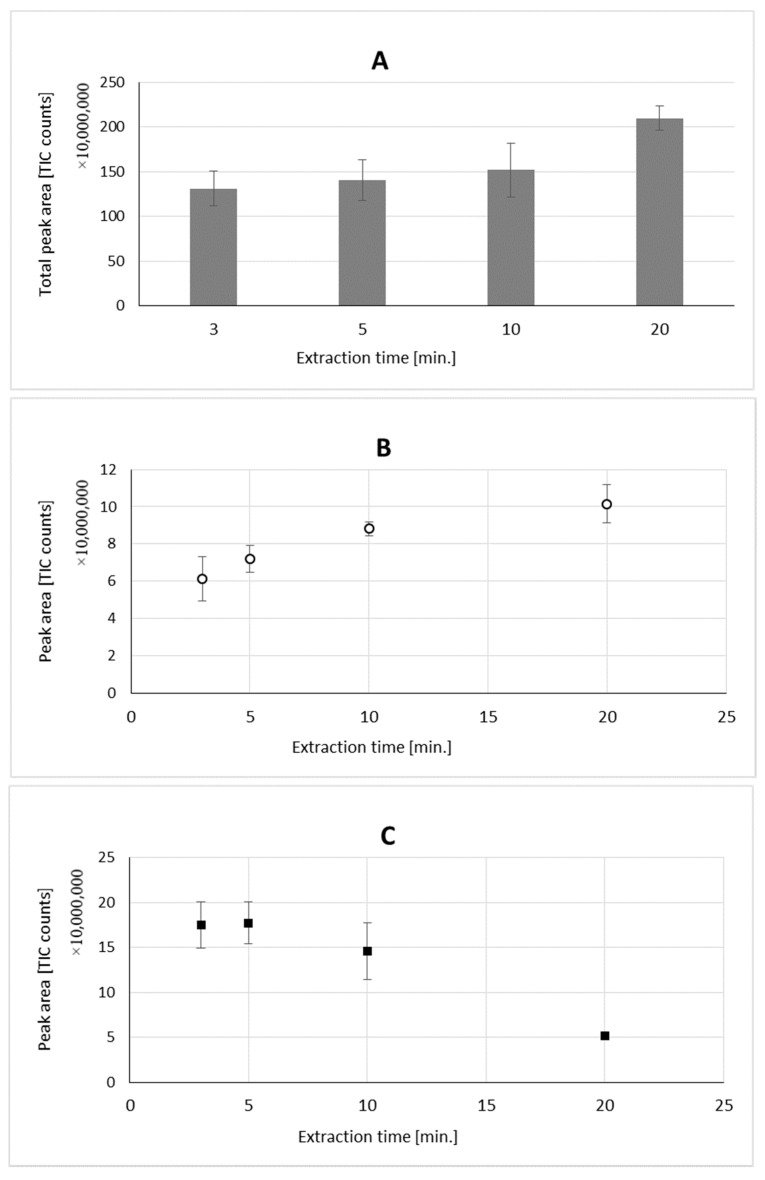
Total peak area of terpenes isolated after 3, 5, 10, and 20 min extraction times (**A**); different extraction behavior of two compounds: camphene (**B**) and γ-terpinene (**C**).

Our approach focuses on implementation of a novel extraction method for isolation of terpenes from plant oil. Extraction of volatile compounds by VASE is an example of sorbent-based extraction, with all of the consequences, including different partition coefficients of analytes and sorbent (Tenax); therefore, different profiles compared to hydrodistillation, and eventual compounds displacement on the surface of the sorbent. Taking into consideration the specificity of the VASE technique, compared to purge and trap the sorbent absorbes analytes in a different way: they are not swept by the stream of inert gas through the sorbent tube, but thanks to the unique construction of Sorbent Pens, they are adsorbed mainly in the initial portion of the sorbent. This allows for rapid desorption without the need for cryofocussing. The sorption process is attained by vacuum, so the air is evacuated from the vial, and after air evacuation, the valve prevents the loss of vacuum. The behavior of compounds in this type of extraction is time dependent. Though the sum of volatiles increased throughout the whole extraction process (comparing 3 min with 20 min, Figure 4A), for different compounds, their amounts adsorbed may be different. For β-pinene, o-cymene, γ-terpinene, linalool, and camphor, their peak areas decreased during extraction (when 3 min and 20 min were compared). For the remaining compounds, their peak areas increased throughout extraction. Figure 4B,C show time related changes in the peak areas of the compounds, which show different extraction profiles (camphene and γ-terpinene). Such behavior of terpenes was also observed in the extraction process using SPME, where polymer based fiber coatings, as well as absorption type of coating (PDMS) were used for the extraction of terpenes from black pepper. It was especially noticeable when the profile of mono- and sesquiterpenes were compared during long extraction times (up to 120 min) [15].

In cold pressed oil from coriander, 17 terpenes were identified. The characteristic flavor of coriander oil is related to the presence of terpenes, as was described earlier [12]. The main compounds responsible for the aroma of *Coriandrum sativum* L. seed essential oil are linalool, α-pinene, terpineol, cuminal, geraniol, and geranyl acetate. In the oil examined by Ravi and coworkers, linalool was the main volatile (57.52%), followed by geranyl acetate (24.51%) [12]. A significant variation in number and concentration of terpenes is noted in literature sources, depending on location, developmental stage, plant part, and overall extraction method.

Coriander essential oil obtained by hydrodistillation (6 h) from seeds (2.2% yield) revealed 53 compounds, where linalool was the main compound (75.30%) [16]. The composition of essential oils changes also with the different parts of coriander fruits. Monoterpene hydrocarbons prevailed in pericarp (26.29%) compared to seed (1.50%), which resulted in 5.41% of monoterpene hydrocarbons in whole fruit. Monoterpene alcohols, which assumed 95.13% in seeds, reached 39.90% in pericarp, which made 89.66% in whole fruit [6]. Msaada and coworkers [17] reported significant changes in the composition of essential oils extracted from the fruits of coriander, depending on the stages of maturity, where linalool was the dominant compound in all ripening stages ranging from 36.69% in the first stage to 72.35% in the fourth stage. Interestingly, geranyl acetate, which amounted 35.17% in the first stage of development dropped down to 1.49% in the last stage [17].

Extraction methods greatly influenced the profile of essential oil. When four extraction techniques were compared for coriander oil (2 h hydrodistillation, 5 h Soxhlet extraction (methylene chloride), 4 h supercritical fluid extraction (SFE, CO_2_), and 20 min subcritical water extraction (SWE) extract yield varied, as other groups of compounds could be co-extracted by some of the methods (Soxhlet, SWE). The content of main compounds—linalool, γ-terpinene, camphor, or geraniol—varied substantially, depending on the technique [9]. Subcritical water extraction compared to hydrodistillation and Soxhlet extraction revealed similar percentages of linalool (82.91%, 77.97%, and 79.62%, respectively,) but for geranyl acetate, varied greatly (0.22%, 2.12%, and 4.36%, respectively) [18]. Microwave assisted hydrodistillation may offer a significant reduction of extraction compared to classic hydrodistillation (60 min vs. 240 min) providing similar profiles of compounds in extracted essential oil [3]. When Schmaus and Kubecka [19] compared lavender oil profiles obtained using hydrodistillation (at pH 7.0) with that of extraction and gas phase stripping, the percentage composition of extracted linalool changed from 40.91% in hydrodistillation to 24.95% and 25.78% for extraction and stripping, respectively. On the other hand, linalyl acetate changed from 13.26% to 42.98% and 40.17% for the mentioned extraction techniques. Some of the compounds were detectable only in hydrodistillation (α-terpineol). As concluded [20], only low and medium molecular weight compounds (up to eicosane in n-alkane series) could be analyzed by gas phase stripping.

Hydrodistillation provides volatile compounds representing essential oils devoid of non-volatile constituents that might be transferred to samples during classical liquid/liquid or liquid/solid extraction. During prolonged (2–6 h) boiling in water, essential oils are evaporated and either directly trapped in organic solvent (simultaneous distillation extraction) or condensed as total oil. Thermal stability of essential oil constituents is an important issue, as terpenes may undergo various changes during prolonged heating. Therefore, hydrodistillation is not the most appropriate isolation method for some plant material (citrus fruit, flowers). As terpenes are unsaturated compounds, they may undergo changes during prolonged boiling. This refers especially to monoterpene alcohols, such as linalool, nerol, geraniol, and α-terpineol. As demonstrated by Rettberg et al. [21] linalool during hydrodistillation can form several pyran and furan oxidized derivatives. In addition, terpene alcohols are prone to acid catalyzed isomerization, and linalool can yield α-terpineol. Moreover, geraniol can be transformed to nerol (and vice versa) and to linalool. In a steam distillation model, a decrease in linalool was noted, with subsequent hydroxyl-linalool derivatives and linalool oxides formation. No such derivatives were observed by us in hydrodistilled coriander oil, though linalool was the main terpene detected in it.

Interestingly, during hydrodistillation, geraniol and geranyl acetate were detected in high amounts, representing 2.13% and 2.68% of total volatiles, respectively (Table 1). No experiments were run on the influence of distillation time on recovery of these compounds. From data obtained for 60 °C VASE extraction compared to 20 °C extraction, it is clear that, to obtain higher relative amounts of these two compounds, an increase of VASE extraction temperature would be needed. On the other hand, increased temperature reduces vacuum in the vial, also influencing the extraction process.

The main terpene alcohol in both extraction types, hydrodistillation and VASE, was linalool. When one compares basic physical parameters for linalool, geraniol, and geranyl acetate, the boiling points are 198 °C, 227 °C, and 238 °C, respectively. Their vapor pressures are 0.16 mm Hg, 0.03 mmHg, and 0.02 mmHg, respectively. Their logP values are 2.97, 3.56, and 4.04, respectively. Finally, the Henry’s constants are: 2.15 × 10^−5^, 1.15 × 10^−5^, and 2.43 × 10^−3^ atm–m^3^/mol respectively. The big differences in vapor pressure are mainly responsible for the low efficiency in VASE extraction, especially performed at 20 °C. Additionally, compounds with logP > 3 are definitely lipophilic, so, in case of geraniol, and even more, geranyl acetate, their migration from lipophilic matrix (oil) to headspace is restricted. It has to be remembered that physical–chemical properties of the oil matrix is a main factor influencing migration of terpenes to the headspace.

## 4. Materials and Methods

### 4.1. Materials and Reagents

Coriander oil was obtained from GAL (Poznań, Poland), which specializes in production of cold pressed oils for pharmaceutical and nutraceutical purposes. The oil was pressed in the GAL factory from coriander fruit, a week before experiments were carried out. Standards of terpenes and fatty acids used in the experiments were purchased from Sigma-Aldrich (Sigma Aldrich, Poznan, Poland). Basic, chemical characteristics of coriander oil were determined in the GAL laboratory (peroxide value (PV), fatty acids methyl esters (FAME) profile).

### 4.2. Analytical Equipment

Terpenes were extracted from oil using the VASE 5800 system (Entech Instruments, Simi Valley, CA, USA). The system is based on extraction of volatile and semivolatile compounds into a trap filled with adsorbent (usually Tenax) with the aid of vacuum. The trap (sorbent pen) has a special construction that allows evacuation of air using a vacuum pump from the vessel/vial in which sampling is performed. After extraction, the vacuum from the vial is released, and the trap is inserted into a special thermal desorber, being part of the system, mounted as an injection port in GC/MS and controlled by the instrument’s electronic pressure control (EPC). The system, apart from thermal desorber, consisted of a sorbent pen conditioner, controller, software for desorption process control, and membrane pump providing a vacuum of 28” Hg. Sorbent pens used in the experiment were filled with Tenax. VASE 5800 system was mounted on a single quadrupole GC/MS system (7890A/7895 TAD MSD, Agilent Technologies, Santa Clara, CA, USA) equipped with a DB-5MS column (30 m × 0.25 mm × 0.50 µm, Agilent Technologies). Helium at the flow of 1 mL/min was used as a carrier gas. The following GC oven program was used for analyses: initial temperature 40 °C for 3 min, then, an increase of 10 °C/min to 180 °C, and 20 °C/min to 280 °C (kept for 4 min). GC/MS transfer line was kept at 290 °C. Mass spectrometer was working in full scan mode in an *m*/*z* range 33–333, 6.6 scans/s. All peak area comparisons, as well as quantitation, was performed for total ion current (TIC) peaks. Terpenes were identified based on comparison with NIST 02 library, and for selected compounds, by comparison with authentic standards. Mass Hunter version 07.00.0 was used for controlling GC/MS system. The following parameters were used for VASE 5800 desorption system: preheat; duration: 2 min at 260 °C. Injection; split (1:25) achieved by splitter frit. Desorption; standby temperature 70 °C, duration 15 min desorption temperature 260 °C, desorption time 20 min. Bakeout; time 5 min at 260 °C. Post bake; duration 4 min, temperature 70 °C. 

For the analysis of essential oil obtained by hydrodistillation, the same column was mounted in a split/splitless injector, and oil was injected manually (0.5 µL) in a split mode (1:100). Analytical parameters for the column and MS were the same as for the VASE extraction.

### 4.3. Sample Preparation and VASE Extraction

Coriander oil was weighted into 44 mL screw top amber vials; the vials were capped with Teflon lined caps. Before analysis, the Teflon lined caps were replaced by special Entech metal caps, which could seal the sorbent pen for sampling. After inserting the sorbent pen into the vial, air was evacuated from the vial via the sorbent pen using a membrane pump with a manometer. Once the vacuum in a vial reached 28” Hg (after approximately 15 s), the pump was disconnected and extraction of volatile compounds began. When needed, the vials were heated in a metal block. Different sample sizes were tested (2000 mg, 1000 mg and 200 mg), as well as different extraction temperatures (60 °C, 40 °C, and 20 °C), and extraction times (3 min, 5 min, 10 min, and 20 min). Quantitative analysis was performed using refined rapeseed oil as a matrix into which terpene standards were dissolved directly (approximately 200 mg per compound in 20 mL of oil); then, serial dilutions were prepared.

### 4.4. Isolation of Essential Oil from Coriander Cold-Pressed Oil

Coriander essential oil was obtained using a traditional hydrodistillation method, using a Deryng apparatus. To a 1 L round flask, 400 mL of water was added and 100 mL of coriander cold pressed oil. The flask was heated and boiling water provided steam to distill off essential oil from cold pressed oil. A total of 7.8 mL of essential oil was collected after 2 h distillation.

## 5. Conclusions

VASE extraction provides rapid characterization of terpene compounds in cold pressed coriander oil, attainable at room temperature within 5 min. To increase the volatiles transfer from the oil matrix to headspace, extraction at higher temperatures (60 °C) can be performed if needed. As the method is partition coefficient dependent, the profile of extracted compounds may differ from hydrodistillation, especially considering proportions of monoterpene alcohols (higher % in distilled oil) and monoterpene hydrocarbons (higher % obtained for VASE extractions). Apart from rapid profiling of terpenes, VASE can be used for their quantitation in oil matrix. The VASE technique relies on a relatively simple setup, in which sorbent pens are desorbed manually, but with satisfying repeatability. Moreover, it does not need cryofocussing, or another type of refocusing in the desorption process, which makes the setup uncomplicated and robust. The setup of the VASE unit and sequence of operations is presented in Supplementary Figure S5. We should also note that sorbent pens can be used multiple times (>hundred from our experience), so single analysis cost is reasonable. This extraction technique is solventless; therefore, contributes to the green chemistry trend, minimizing its impact on the environment.

## Figures and Tables

**Table 1:** Essential oil constituents identified in cold-pressed coriander oil.

No.	Rt	RI DB-5	Name	CAS	Hydr. (%)	VASE 20 °C (%)	VASE 60 °C (%)
1	11.252	926	β-thujene	28634-89-1	0.24	0.81	0.61
2	11.489	938	α-pinene *	7785-70-8	12.79	17.94	11.99
3	11.830	956	(-)-camphene *	79-92-5	2.32	4.12	3.55
4	12.185	974	Sabinene	3387-41-5	0.82	1.24	1.65
5	12.361	982	β-pinene *	127-91-3	3.01	9.70	9.39
6	12.802	1007	α-phellandrene	99-83-2	0.05	0.36	0.62
7	12.989	1020	α-terpinene *	99-86-5	0.07	0.46	0.76
8	13.136	1026	o-cymene	527-84-4	3.16	8.92	7.76
9	13.247	1034	Sylvestrene	1461-27-4	4.34	10.10	8.94
10	13.310	1042	Unknown		0.46	0.69	0.77
11	13.373	1045	β-cis ocimene	3338-55-4	0.32	1.47	3.34
12	13.732	1062	γ-terpinene *	99-85-4	9.47	13.96	12.56
13	14.205	1088	Terpinolene *	586-62-9	0.93	1.93	2.53
14	14.363	1100	β-linalool *	78-70-6	44.98	23.05	27.69
15	14.769	1129	allo-ocimene	7216-56-0	0.09	0.72	0.98
16	15.404	1166	Camphor *	76-22-2	6.26	3.92	4.58
17	16.018	1207	Unknown		0.39	0.11	0.31
18	16.681	1248	Geraniol *	106-24-1	2.13	0.27	1.04
19	18.322	1371	Geranyl acetate	105-87-3	2.68	0.23	0.93
20	21.703	nd	Unknown		2.70	0.00	0.00
21	25.853	nd	unknown		2.78	0.00	0.00

Rt: retention time (min); RI DB-5: retention indices determined for DB-5 column; CAS–Hydr. (%): percentage (*w*/*w*) of compounds in essential oil obtained after hydrodistillation (2 h); VASE 20 °C (%): percentage (*w*/*w*) of compounds in essential oil obtained after VASE extraction at 20 °C for 5 min; VASE 60 °C (%): percentage (*w*/*w*) of compounds in essential oil obtained after VASE extraction at 60 °C for 5 min. Asterisk (*) denotes compounds identified by comparison of their chromatographic parameters and mass spectra with authentic standards. nd—not determined.

## Data Availability

Data is contained within the article and supplementary files.

## Supplementary Materials

The following are available online, Figure S1: Chromatograms of volatile compounds extracted from coriander oil samples of different weight using VASE; A—2000 mg; B—1000 mg; C—200 mg, Figure S2: Chromatograms of volatile compounds extracted from coriander oil samples at different temperatures using VASE; A—20 °C; B—40 °C; C—60 °C, Figure S3: Chromatograms of volatile compounds extracted from coriander oil samples using different extraction time and VASE; A—3 min; B—5 min; C—10 min; D—20 min, Figure S4: Standard curves of different types used for quantitation of terpenes by VASE: A—camphor; B—α-terpinene, Figure S5: scheme of VASE analytical setup, together with sorbent pens and vials with caps designed for VASE extraction.

## Sample Availability

Samples of the compounds are not available from the authors.
