# Supplementary material for: Determination of Volatile Terpenes in Coriander Cold Pressed Oil by Vacuum Assisted Sorbent Extraction (VASE)
Source: Molecules. 2021 Feb 8;26(4):884. doi: 10.3390/molecules26040884 (PMC7915774; doi:10.3390/molecules26040884)
Supplement: Supplementary file 1 [file molecules-26-00884-s001.pdf]

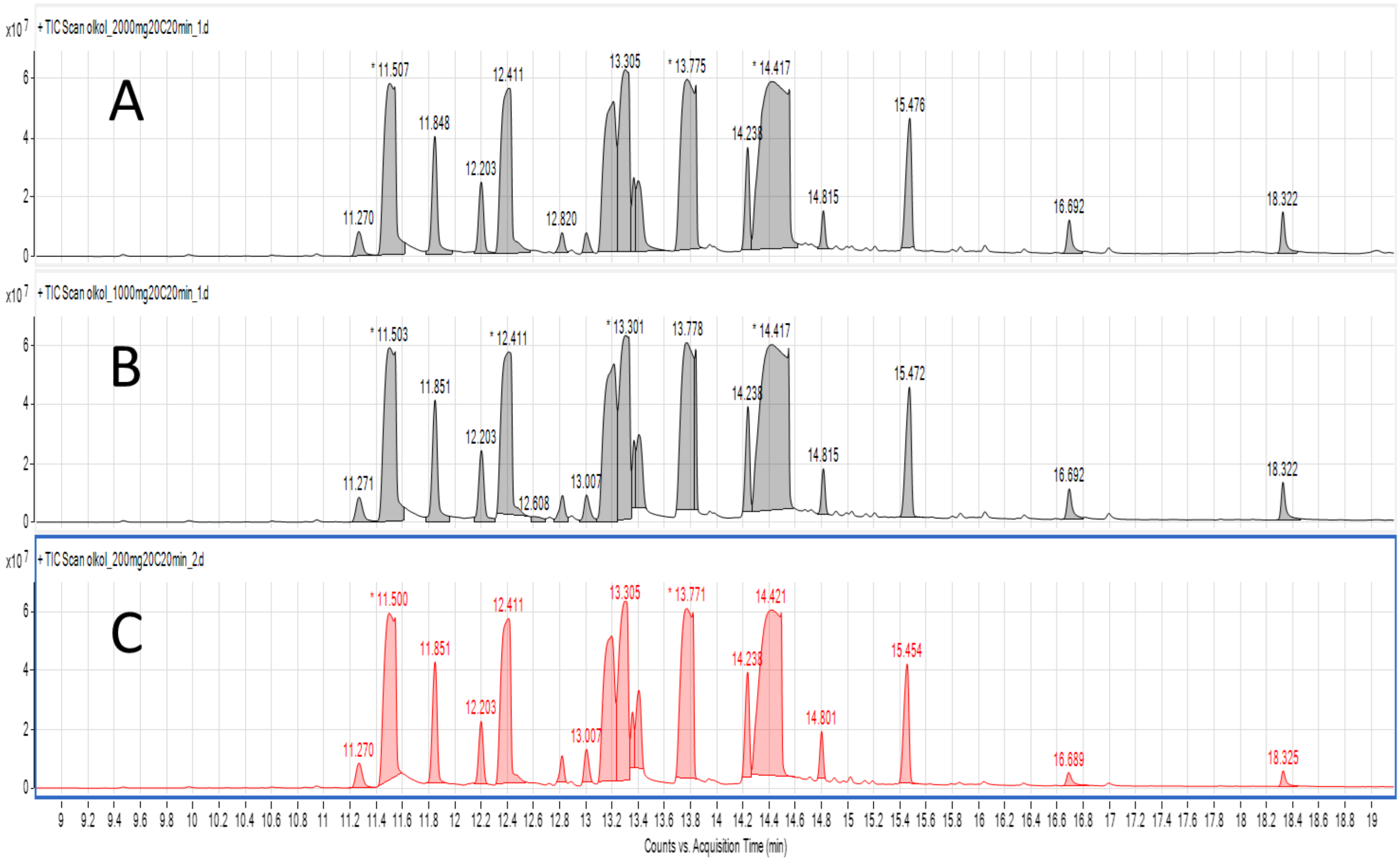

Figure 1S. Chromatograms of volatile compounds extracted from coriander oil samples of different weight using VASE; **A** – 2000mg; **B** – 1000mg; **C** – 200mg. All extractions performed at 60°C for 20 min. TIC chromatogram in red indicates parameter chosen for further experiments.

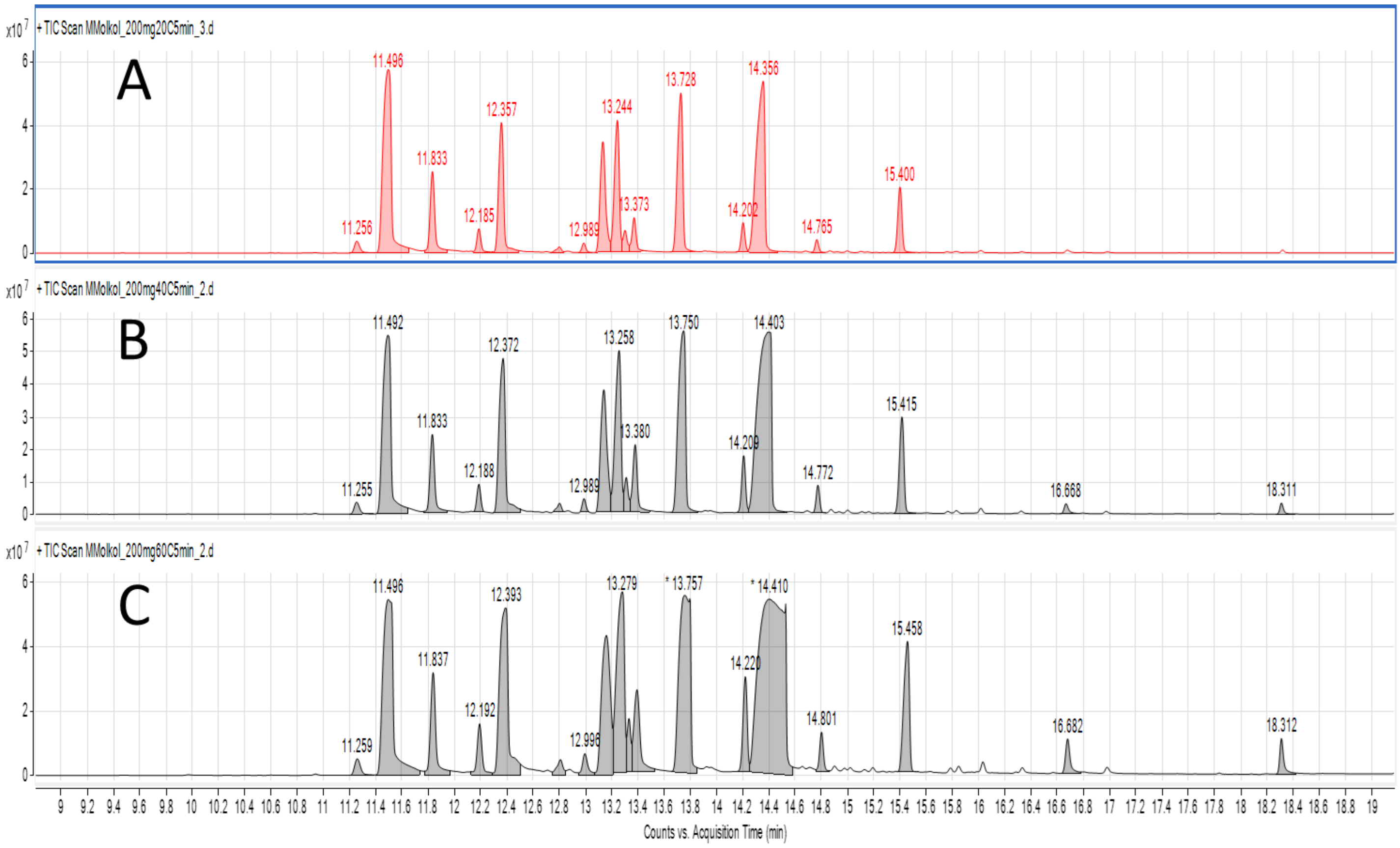

Figure 2S. Chromatograms of volatile compounds extracted from coriander oil samples at different temperatures using VASE; **A** – 20°C; **B** – 40°C; **C** – 60°C. All extractions performed for 20 min. using sample size of 200mg. TIC chromatogram in red indicates parameter chosen for further experiments.

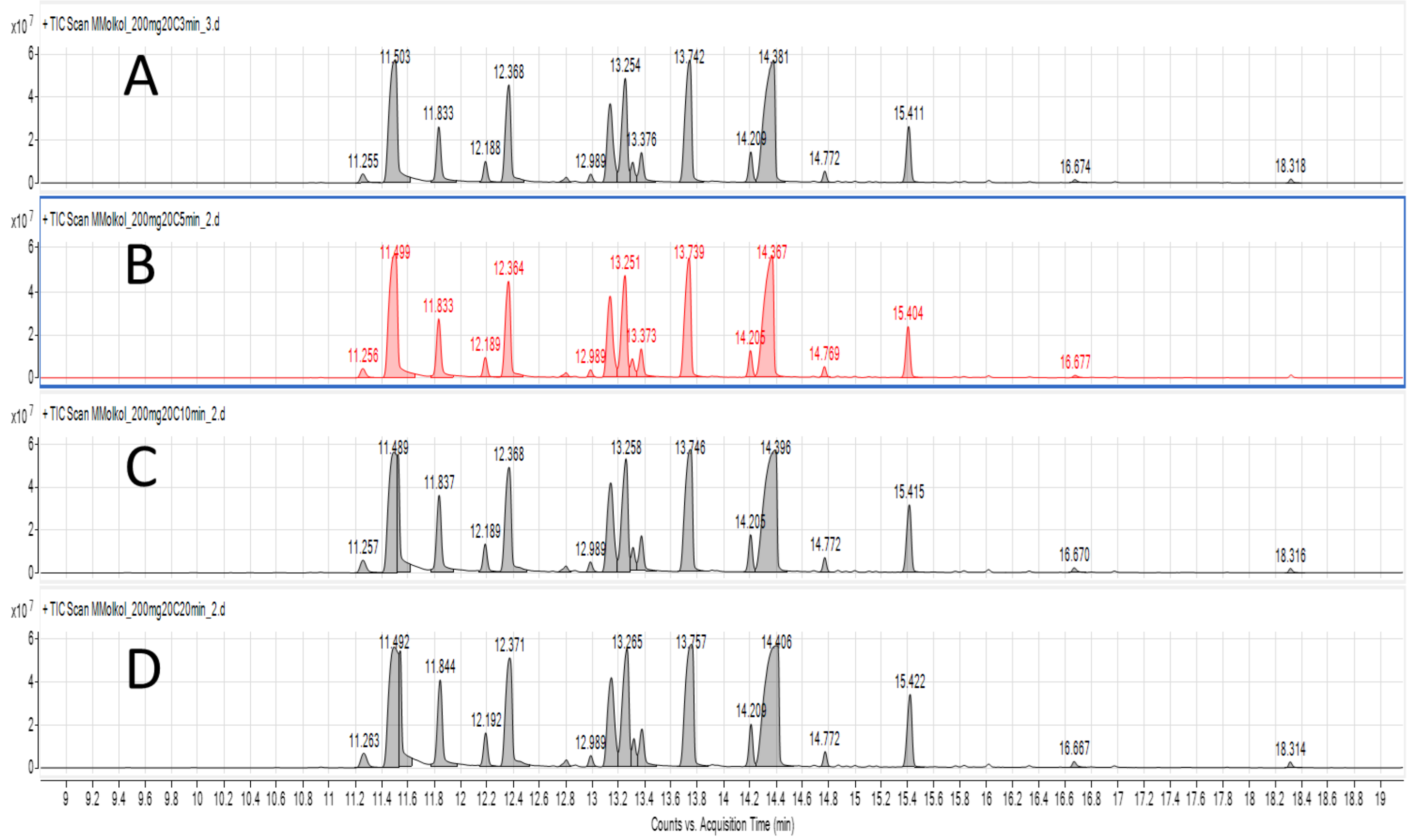

Figure 3S. Chromatograms of volatile compounds extracted from coriander oil samples using different extraction time and VASE; **A** – 3 min.; **B** – 5 min.; **C** – 10min.; **D** – 20 min. All extractions performed at 20°C using 200mg oil sample. TIC chromatogram in red indicates parameter chosen for further experiments.

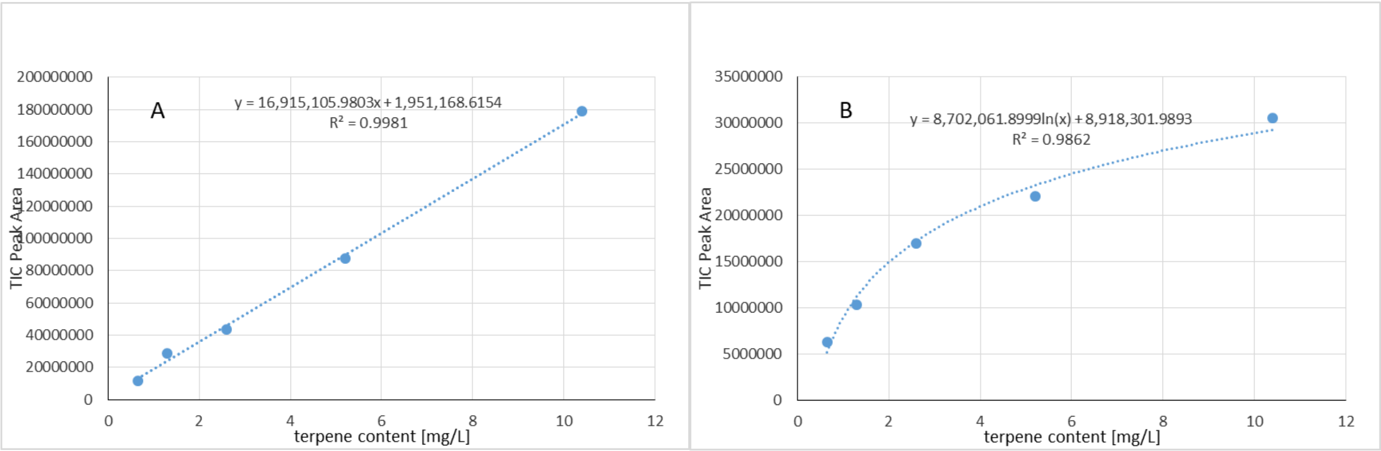

Figure 4S. Standard curves of different types used for quantitation of terpenes by VASE: A – camphor; B – α-terpinene.

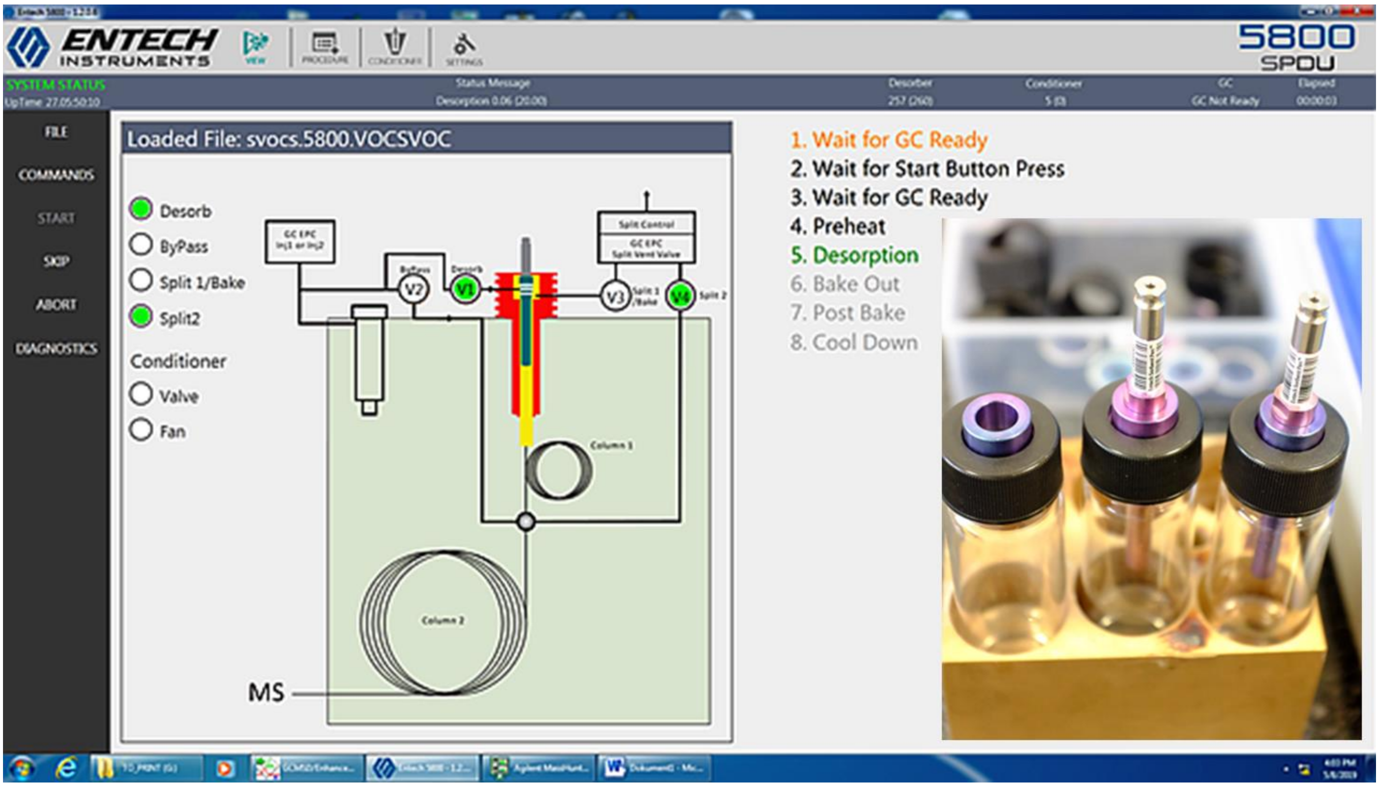

Figure 5S. Scheme of VASE analytical setup (shown in control panel of ENTECH 5800 desorber) together with sorbent pens and vials with caps designed for VASE extraction
